# Prevalence of Deleterious Variants in *MC3R* in Patients With Constitutional Delay of Growth and Puberty

**DOI:** 10.1210/clinem/dgad373

**Published:** 2023-06-20

**Authors:** Katie Duckett, Alice Williamson, John W R Kincaid, Kara Rainbow, Laura J Corbin, Hilary C Martin, Ruth Y Eberhardt, Qin Qin Huang, Matthew E Hurles, Wen He, Raja Brauner, Angela Delaney, Leo Dunkel, Romina P Grinspon, Janet E Hall, Joel N Hirschhorn, Sasha R Howard, Ana C Latronico, Alexander A L Jorge, Ken McElreavey, Verónica Mericq, Paulina M Merino, Mark R Palmert, Lacey Plummer, Rodolfo A Rey, Raíssa C Rezende, Stephanie B Seminara, Kathryn Salnikov, Indraneel Banerjee, Brian Y H Lam, John R B Perry, Nicholas J Timpson, Peter Clayton, Yee-Ming Chan, Ken K Ong, Stephen O’Rahilly

**Affiliations:** Wellcome-MRC Institute of Metabolic Science, Box 289, Level 4, Addenbrooke’s Hospital, Cambridge CB2 0QQ, UK; Wellcome-MRC Institute of Metabolic Science, Box 289, Level 4, Addenbrooke’s Hospital, Cambridge CB2 0QQ, UK; Wellcome-MRC Institute of Metabolic Science, Box 289, Level 4, Addenbrooke’s Hospital, Cambridge CB2 0QQ, UK; Wellcome-MRC Institute of Metabolic Science, Box 289, Level 4, Addenbrooke’s Hospital, Cambridge CB2 0QQ, UK; MRC Integrative Epidemiology Unit, University of Bristol, Oakfield House, Oakfield Grove, Bristol BS8 2BN, UK; Human Genetics, Wellcome Sanger Institute, Wellcome Genome Campus, Hinxton, Cambridge CB10 1SA, UK; Wellcome Sanger Institute, Wellcome Genome Campus, Hinxton, Cambridge CB10 1SA, UK; Human Genetics, Wellcome Sanger Institute, Wellcome Genome Campus, Hinxton, Cambridge CB10 1SA, UK; Wellcome Sanger Institute, Wellcome Genome Campus, Hinxton, Cambridge CB10 1SA, UK; Division of Endocrinology, Department of Pediatrics, Boston Children’s Hospital, 300 Longwood Ave, Boston, MA 02115, USA; Pediatric Endocrinology Unit, Hôpital Fondation Adolphe de Rothschild and Université Paris Cité, 25 rue Manin, 75019 Paris, France; Division of Endocrinology, Department of Pediatric Medicine, St. Jude Children’s Research Hospital, 262 Danny Thomas Place MS 737, Memphis, TN 38105, USA; Centre for Endocrinology, William Harvey Research Institute, Barts & the London Medical School, Charterhouse Square, London EC1M 6BQ, UK; Centro de Investigaciones Endocrinolègicas “Dr. César Bergadá” (CEDIE), CONICET–FEI–Divisièn de Endocrinología, Hospital de Niños Ricardo Gutiérrez, Gallo 1330, C1425EFD Buenos Aires, Argentina; Clinical Research Branch, Division of Intramural Research, National Institute of Environmental Science, National Institute of Health, 111 TW Alexander Dr, Bldg 101 – A222, Research Triangle Park, NC 27709, USA; Division of Endocrinology, Department of Pediatrics, Boston Children’s Hospital, 300 Longwood Ave, Boston, MA 02115, USA; Centre for Endocrinology, William Harvey Research Institute, Queen Mary University of London, Charterhouse Square, London EC1M 6BQ, UK; Departamento de Clínica Médica, Av. Dr. Arnaldo, 455 - Cerqueira César, 01246903 São Paulo - SP, Brazil; Departamento de Clínica Médica, Av. Dr. Arnaldo, 455 - Cerqueira César, 01246903 São Paulo - SP, Brazil; Institut Pasteur, Université de Paris, CNRS UMR3738, Human Developmental Genetics, F-75015 Paris, France; Institute of Maternal and Child Research, Faculty of Medicine, University of Chile, Santa Rosa 1234, 2° piso, Santiago 8320000, Chile; Institute of Maternal and Child Research, Faculty of Medicine, University of Chile, Santa Rosa 1234, 2° piso, Santiago 8320000, Chile; Division of Endocrinology, The Hospital for Sick Children and Departments of Pediatrics and Physiology, University of Toronto, Toronto, ON M5G 1X8, Canada; Massachusetts General Hospital Harvard Center for Reproductive Medicine and Reproductive Endocrine Unit, Massachusetts General Hospital, Bartlett Hall Extension, 5th Floor, 55 Fruit Street, Boston, MA 02114, USA; Centro de Investigaciones Endocrinolègicas “Dr. César Bergadá” (CEDIE), CONICET–FEI–Divisièn de Endocrinología, Hospital de Niños Ricardo Gutiérrez, Gallo 1330, C1425EFD Buenos Aires, Argentina; Departamento de Clínica Médica, Av. Dr. Arnaldo, 455 - Cerqueira César, 01246903 São Paulo - SP, Brazil; Massachusetts General Hospital Harvard Center for Reproductive Medicine and Reproductive Endocrine Unit, Massachusetts General Hospital, Bartlett Hall Extension, 5th Floor, 55 Fruit Street, Boston, MA 02114, USA; Massachusetts General Hospital Harvard Center for Reproductive Medicine and Reproductive Endocrine Unit, Massachusetts General Hospital, Bartlett Hall Extension, 5th Floor, 55 Fruit Street, Boston, MA 02114, USA; Department of Paediatric Endocrinology, Royal Manchester Children’s Hospital, Manchester M13 9WL, UK; Wellcome-MRC Institute of Metabolic Science, Box 289, Level 4, Addenbrooke’s Hospital, Cambridge CB2 0QQ, UK; Wellcome-MRC Institute of Metabolic Science, Box 289, Level 4, Addenbrooke’s Hospital, Cambridge CB2 0QQ, UK; MRC Integrative Epidemiology Unit, University of Bristol, Oakfield House, Oakfield Grove, Bristol BS8 2BN, UK; Paediatric Endocrinology, Royal Manchester Children’s Hospital, Oxford Road, Manchester M13 9WL, UK; Division of Endocrinology, Department of Pediatrics, Boston Children’s Hospital, 300 Longwood Ave, Boston, MA 02115, USA; MRC Epidemiology Unit, Institute of Metabolic Science, Cambridge Biomedical Campus Box 285, University of Cambridge, Cambridge CB2 0QQ, UK; Wellcome-MRC Institute of Metabolic Science, Box 289, Level 4, Addenbrooke’s Hospital, Cambridge CB2 0QQ, UK

**Keywords:** delayed puberty, constitutional delay, ALSPAC, UK Biobank, *MC3R*

## Abstract

**Context:**

The melanocortin 3 receptor (MC3R) has recently emerged as a critical regulator of pubertal timing, linear growth, and the acquisition of lean mass in humans and mice. In population-based studies, heterozygous carriers of deleterious variants in *MC3R* report a later onset of puberty than noncarriers. However, the frequency of such variants in patients who present with clinical disorders of pubertal development is currently unknown.

**Objective:**

This work aimed to determine whether deleterious *MC3R* variants are more frequently found in patients clinically presenting with constitutional delay of growth and puberty (CDGP) or normosmic idiopathic hypogonadotropic hypogonadism (nIHH).

**Methods:**

We examined the sequence of *MC3R* in 362 adolescents with a clinical diagnosis of CDGP and 657 patients with nIHH, experimentally characterized the signaling properties of all nonsynonymous variants found and compared their frequency to that in 5774 controls from a population-based cohort. Additionally, we established the relative frequency of predicted deleterious variants in individuals with self-reported delayed vs normally timed menarche/voice-breaking in the UK Biobank cohort.

**Results:**

*MC3R* loss-of-function variants were infrequent but overrepresented in patients with CDGP (8/362 [2.2%]; OR = 4.17; *P* = .001). There was no strong evidence of overrepresentation in patients with nIHH (4/657 [0.6%]; OR = 1.15; *P* = .779). In 246 328 women from the UK Biobank, predicted deleterious variants were more frequently found in those self-reporting delayed (aged ≥16 years) vs normal age at menarche (OR = 1.66; *P* = 3.90E-07).

**Conclusion:**

We have found evidence that functionally damaging variants in *MC3R* are overrepresented in individuals with CDGP but are not a common cause of this phenotype.

Delayed puberty (DP) is a common clinical presentation that often causes psychological distress to affected individuals and is associated with adverse health outcomes later in life (1, 2). Its most common form is self-limited DP, in which pubertal onset occurs at the extreme end of normal and before age 18 years and is followed by normal reproductive hormone activity (3). This pattern may be accompanied by reduced linear growth that is already apparent from early childhood (“constitutional delay of growth and puberty,” CDGP). In contrast, individuals with hypogonadotropic hypogonadism (HH) are unable to progress through puberty without hormone replacement therapy. Self-limited DP is often difficult to distinguish from HH during adolescence. Therefore, understanding the etiology may allow the earlier diagnosis of DP and HH, as well as inform the development of new treatments (4).

DP is highly heritable, based on analysis of affected pedigrees. The pattern of inheritance is often autosomal dominant with incomplete penetrance (5-9). Rare variants in genes that regulate hypothalamic-pituitary function and embryonic migration of gonadotrophin-releasing hormone neurons have been identified in patients with HH (8-12). Furthermore, genome-wide association studies in large population-based cohorts have identified hundreds of common variants associated with normal variation in puberty timing both in males and females (13, 14), and these also implicate many genes involved in the development and functioning of the hypothalamic-pituitary-gonadal axis.

Protein-altering variants in the melanocortin 3 receptor gene (*MC3R*) were recently shown to confer later puberty timing in males and females from UK population-based cohorts, and a single male who was homozygous for a severely disruptive variant entered puberty after age 20 years (15). *MC3R* is highly expressed in the KNDy neurons of the hypothalamic arcuate nucleus, which produce kisspeptin, the key central activator of the sex hormone axis (15-18). We proposed that MC3R signaling via the leptin-POMC-melanocortin pathway is a permissive signal that activates pubertal hormone activity in response to adequate nutritional status (15).

Here, we hypothesized that deleterious *MC3R* variants might also be implicated in the etiology of CDGP. To test this hypothesis, we first experimentally characterized all *MC3R* protein-coding variants that have not been previously reported in a population-based cohort, the Avon Longitudinal Study of Parents and Children (ALSPAC). We then further experimentally characterized all *MC3R* protein-coding variants in 2 cohorts of patients with CDGP and a cohort of patients with normosmic idiopathic HH (nIHH) and used these results to compare the frequency of deleterious variants to the background population. Finally, we set out to determine whether individuals at the extreme ends of self-reported pubertal timing within the general population are more likely to carry a deleterious *MC3R* variant, using the UK Biobank population-based cohort.

## Materials and Methods

### Patient Cohorts and Genetic Sequencing

Three cohorts of patients were examined:

- 278 individuals (169 males) of diverse ancestries clinically diagnosed with CDGP from the Delayed Puberty Genetic Consortium (DPGen) for whom whole-exome sequence (WES) data were available (with sequencing performed as described in Zhu et al (8)). Inclusion criteria for a CDGP diagnosis are as described in Jonsdottir-Lewis et al (19), with the additional criterion that patients did not have a relative diagnosed with IHH.
- 84 individuals (80 males) of majority European ancestry clinically diagnosed with CDGP in Manchester, UK (20), in whom targeted sequencing of *MC3R* was performed. Full inclusion criteria are described in Banerjee et al (20).
- 657 individuals clinically diagnosed with nIHH (aged ≥18 years, sex steroids below the adult reference range, gonadotropins not elevated above the reference range). Patients with nIHH were enrolled in a genetic study at Massachusetts General Hospital as previously described (21). WES was performed using the Broad Institute Genomics platform in the 657 patients, and variant-calling was performed as previously described (22).

All human participant research was approved by the ethics boards of each institution, and written informed consent was obtained for all study participants.

### Population Cohorts and Genetic Sequencing

#### Avon Longitudinal Study of Parents and Children cohort

Individuals from generation 1 (G1) of the UK Avon Longitudinal Study of Parents and Children (ALSPAC) birth cohort study were included as an unselected population cohort of majority European descent (23-25). Ethical approval for the study was obtained from the ALSPAC Ethics and Law Committee and the Local Research Ethics Committees. Consent for biological samples has been collected in accordance with the Human Tissue Act (2004).

Two sources of genetic data were used in the estimation of *MC3R* deleterious variant frequencies. First, a pooled high-throughput sequencing approach was used to identify all nonsynonymous coding variants in *MC3R* in 5774 individuals for whom DNA was available (as described in Lam et al (15)). Briefly, 5992 individual DNA samples (representing 5774 unique individuals) were combined into pools of 50 and sequenced at the *MC3R* locus. Individual DNA present in pools containing 3 identified complete loss-of-function (CLoF) variants were Sanger-sequenced to determine the number of carriers of CLoF variants. Second, to determine the frequency of a partial loss-of-function (PLoF) missense mutation in *MC3R*, R220S (rs61735259), this variant was assessed in the wider ALSPAC cohort using a combination of WES data (available for 8301 participants) and Haplotype Reference Consortium (HRC)-imputed data (available for an additional 1665 participants). A description of the derivation and quality control of WES data can be found in the Supplementary Materials (26).

High concordance was observed between carriers identified in the ALSPAC HRC-imputed data set and carriers identified in the WES data (Supplementary Table S1 (26)). All individuals who were labeled as carriers in the HRC data set had this status confirmed in the WES data (where they existed). Given high concordance, genotype data were combined to give a maximized final genotype designation.

#### UK Biobank cohort

A total of 436 865 unrelated individuals (246 328 women and 190 547 men) of majority European descent for whom age at menarche or voice-breaking data were available were included in this analysis (27). WES data for these individuals underwent variant-level quality control as described in Gardner et al (28). No other exclusion criteria were applied.

### Sequencing the *MC3R* Locus

For 90 CDGP patients without WES data, DNA was amplified in the *MC3R* coding region and Sanger-sequenced as detailed in Lam et al (15). For full coverage of the *MC3R* coding sequence, the following primers were used:

- MC3R_F1: 5′-TGGAACAGCAAAGTTCTCCCT-3′
- MC3R_R3: 5′-CCTCACGTGGATGGAAAGTC-3′
- MC3R_2F: 5′-CAGCATCATGACCGTGAGGAA-3′
- MC3R_1R: 5′-CGAAGGTCAGGTAGTCGCTG-3′
- MC3R_2R: 5′-TGCATGAGTGTTGCTGTGGG-3′

### Functional Assays for Cyclic Adenosine Monophosphate Activity

*MC3R* coding variants were cloned into a wild-type (WT) human N-FLAG-MC3R pcDNA3.1(+) vector using site-directed mutagenesis (Agilent Quikchange Lightning kit). HEK293 cells (ATCC) were plated into a 96-well plate (Greiner) at a density of 20 000 cells per well and transfected with 10 ng WT or variant construct in duplicate using Lipofectamine 3000 (Invitrogen) as per the manufacturer's protocol. After 48 hours, cells were treated with a final concentration range of 10^−14^ to 10^−5^M NDP-α-MSH (Bachem) in phosphate-buffered saline for 2 hours. Cyclic adenosine monophosphate (cAMP) levels were measured using the Eurofins DiscoverX Hithunter cAMP assay for Small Molecules as per the manufacturer's protocol. Luminescence readings were taken on a Tecan M1000 Pro plate reader.

For all constructs, raw luminescence values were converted to cAMP concentration (μM) using a cAMP standard curve (∼0-10 μM) and 3-parameter dose-response curves were fitted using GraphPad Prism v.6.0 to calculate V_max_ and logEC_50_ values for WT variants. All values were normalized to WT V_max_ (100%) and replotted for LoF determination. For both V_max_ and logEC_50_ nonnormalized values, statistical difference from WT was determined using 2-way ANOVA for all variants except I50T, with variant, plate number, and day of assay as covariates in the model, followed by post hoc Dunnett test to determine statistical significance for each variant (calculated using R v4.1.3). I50T was analyzed separately using the same workflow as it was assayed at a different time separately from all the other variants.

Variants were classified as WT-like (75% WT < V_max_ ≤ 120% WT or 0.2 × WT ≤ EC_50_ < 5 × WT), PLoF (25% WT < V_max_ ≤ 75% WT or 5 × WT ≤ EC_50_ < 50 × WT), or CLoF (V_max_ ≤ 25% WT or EC_50_ ≥ 50 × WT).

### Odds Ratios Calculations in Patient Cohorts

The number of carriers of functionally characterized *MC3R* LoF variants was counted for each of the patient cohorts and the unselected ALSPAC cohort. Exact odds ratios (ORs) for carrying PLoF, CLoF, or combined PLoF/CLoF were calculated in the patient cohorts compared to ALSPAC and *P* values calculated using the Fisher exact test (*fisher.test()* function in base R *stats* package v4.1.0).

### Odds Ratios Calculations in UK Biobank

*MC3R* protein-altering variants present in the UK Biobank (n = 171) were grouped into variant classes according to their bioinformatically predicted functional impact:

- High = predicted high impact by variant effect predictor (VEP; v104) (premature stop variants, frameshift variants, start-lost variants, stop-lost variants) (29).
- Moderate excl. benign = variants predicted as moderate impact by VEP (missense/inframe insertions/deletions) excluding those predicted “benign” by SIFT or “non-damaging” by PolyPhen-2 (30, 31).
- Moderate/high excl. benign = variants fitting into the aforementioned 2 classes combined.
- CADD ≥25 = variants with a predicted damaging score over 25 by Combined Annotation-Dependent Depletion (CADD) v1.6 (32).

Women in the UK Biobank self-reported their age at menarche to the nearest whole year (UKB field ID = 2714). We categorized these responses as: delayed (age ≥16 years), early (<10 years), or within the typical range—defined as “normal” (age 10-15 inclusive). Men in the UK Biobank self-reported their age at voice-breaking (UKB field ID = 2385) as “later than average,” “earlier than average,” or “about average” compared to peers. Exact ORs of carriers vs noncarriers were calculated and *P* values were calculated using a Fisher exact test through the use of the *oddsratio()* function in R package *epitools* (v0.5-10.1).

## Results

### Frequency of Deleterious *MC3R* Variants in the Avon Longitudinal Study of Parents and Children

To assess potential overrepresentation of deleterious *MC3R* variants in DP cohorts, we first established the number of carriers of these variants in the population-based ALSPAC birth cohort to indicate their background frequency.

**Figure 1.**
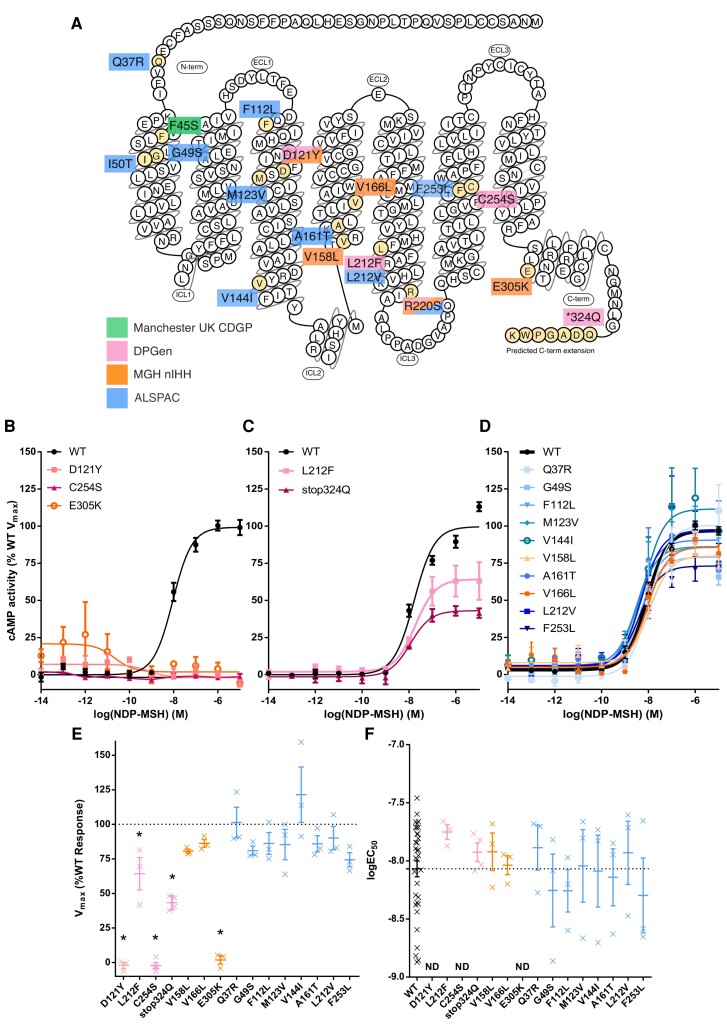
Functional characterization of melanocortin 3 receptor (*MC3R*) coding mutations in constitutional delay of growth and puberty (CDGP) and population cohorts. A, Schematic showing locations of identified nonsynonymous variants in Manchester UK CDGP, normosmic idiopathic hypogonadotropic hypogonadism (nIHH), Delayed Puberty Genetic Consortium (DPGen), and Avon Longitudinal Study of Parents and Children (ALSPAC) cohorts. F45S has already been characterized as complete loss-of-function (CLoF), and R220S as partial loss-of-function (PLoF). B to D, cyclic adenosine monophosphate (cAMP) dose-response curves on treatment with NDP-MSH classified as CLoF, PLoF, and wild-type (WT)-like variants, respectively. Error bars = SEM, N reported in Supplementary Materials. E and F, V_max_ (%WT) and logEC50 values plotted for all variants except I50T (Supplementary Materials) calculated from dose-response curves. Dotted line indicates WT response. Individual crosses represent biological replicates. Asterisk represents Bonferroni *P* less than .05 via 2-way analysis of variance compared to WT. ND, not determined.

We previously reported 20 nonsynonymous coding *MC3R* variants found within 5774 individuals in ALSPAC; 3 variants present in 6 individuals were functionally characterized as CLoF, 1 variant present in an unknown number of individuals was characterized as PLoF, and 7 were WT-like (15). In this work we experimentally characterized the 9 remaining variants found in these individuals to determine if any further LoF variants were present. All 9 variants were categorized as WT-like based on their EC_50_ and V_max_ values (Fig. 1, Supplementary Fig. S1, and Supplementary Table S2 (26)). Overall, we found the frequency of *MC3R* CLoF variant carriers in ALSPAC to be 1.0 out of 1000 participants (Table 1). To determine the number of carriers of the single PLoF variant found in ALSPAC, R220S, we combined WES data with HRC-imputed derived R220S genotypes available for 9966 participants. In total, 42 were designated as PLoF carriers (36 WES and 6 HRC-imputed), giving an overall PLoF carrier frequency of 4.2 out of 1000 participants (see Table 1).

**Table 1.** Frequencies of validated *MC3R* loss of function variants in delayed puberty cohorts

		PLoF					CLoF					Combined PLoF and CLoF				
Cohort	Total No.	No. carriers	Frequency (per 1000)	OR vs ALSPAC	95% CI	*P*	No. carriers	Frequency (per 1000)	OR vs ALSPAC	95% CI	*P*	No. carriers	Frequency (per 1000)	OR vs ALSPAC	95% CI	*P*
Manchester UK CDGP	84	0	0	—	—	*—*	1	11.9	11.44	0.25-95.9	.097	1	11.9	2.25	0.06-13.4	.366
DPGen	278	5	18.0	4.27	1.31-10.9	.009	2	7.2	6.92	0.68-39.0	.050	7	25.2	4.75	1.81-10.6	.001
Total DP:	362	5	13.8	3.28	1.01-8.34	.024	3	8.3	7.97	1.28-37.5	.013	8	22.1	4.17	1.70-8.91	.001
nIHH	657	2	3.0	0.72	0.08-2.79	≥.999	2	3.0	2.93	0.29-16.4	.194	4	6.1	1.15	0.30-3.13	.779
ALSPAC	5774 (9966)	42	4.2	—	—	*—*	6	1.0	—	—	*—*	—	5.3	—	0	*—*

All *MC3R* coding variants were experimentally characterized as PLoF (V_max_ >25% V_max_ [WT] and V_max_ ≤75% V_max_ [WT]) or CLoF (V_max_ ≤25% V_max_ [WT]). ORs, 95% CIs, and *P* values for frequency of LoF mutations compared to ALSPAC controls were calculated using the Fisher exact test. For CLoF variants, data were available for 5774 ALSPAC individuals, and for PLoF variants 9966 ALSPAC individuals. For combined PLoF and CLoF, a combined frequency in ALSPAC was calculated.

Abbreviations: ALSPAC, Avon Longitudinal Study of Parents and Children; CDGP, constitutional delay of growth and puberty; CLoF, complete loss-of-function; DP, delayed puberty; LoF, loss-of-function; *MC3R*, melanocortin 3 receptor; nIHH, normosmic idiopathic hypogonadotropic hypogonadism; OR, odds ratio; PLoF, partial loss-of-function; WT, wild-type.

### Frequency of Deleterious *MC3R* Variants in Constitutional Delay of Growth and Puberty

Of the 362 patients with CDGP (84 from Manchester UK CDGP and 278 from DPGen), 8 patients carried a heterozygous nonsynonymous *MC3R* variant (F45S, D121Y, L212F, R220S, C254S, and a stop-lost variant—stop324Q). C254S and D121Y were functionally characterized as CLoF, and L212F as PLoF (see Fig. 1). F45S has been previously reported as CLoF and R220S as PLoF (15). The patient with the F45S variant had inherited this from his father, who also had a history of DP.

The stop-lost variant (stop324Q) was predicted to lead to an additional 7 amino acids at the C-terminus followed by an alternative stop codon, based on the transcribed 3′ untranslated region sequence (33, 34). This C-terminally extended version of the receptor was cloned and functionally characterized and classified as PLoF (see Fig. 1). However, we cannot be sure that this variant is successfully transcribed and translated in the in vivo context as it may affect messenger RNA and/or protein stability leading to further LoF.

Overall, the frequency of *MC3R* LoF variant carriers was 22.1 out of 1000 patients with CDGP, which is 4.17-fold higher (*P* = .001) than was found in the population-based ALSPAC study (see Table 1).

As 162 of 278 participants within DPGen were of non-European ancestry, we performed a sensitivity analysis limiting the DPGen data set to the 116 individuals of non-Finnish European descent, reflective of the ALSPAC control cohort, and very similar LoF carrier frequency and OR were observed (26.1/1000 patients; OR = 4.66; *P* = .031) (Supplementary Table S3 (26)).

In contrast to patients with CDGP, the frequency of *MC3R* LoF variant carriers among patients with nIHH (6.1/1000) was not largely different from that in controls (Table 1). Two of 657 nIHH patients carried the R220S variant, 2 of 657 patients carried variants functionally characterized as CLoF (D121Y and E305K), and 2 of 657 patients carried variants characterized as WT-like (V158L and V166L) (see Fig. 1 and Supplementary Table S2 (26)).

### *MC3R* Associations With Delayed Puberty in the UK Biobank

Among 246 328 women from the UK Biobank, *MC3R* variant carrier status was associated with higher odds of self-reported delayed menarche (age at menarche ≥16 years), with stronger estimated effects seen for more deleterious variants (Table 2). High-impact variants were 2.3-fold more frequent (*P* = .0098) in individuals with delayed menarche compared to those with a normal age at menarche and predicted deleterious (CADD ≥ 25) variants were 1.7-fold more frequent (*P* = 3.90E-07). In contrast, individuals with early menarche (age <10 years) were less likely to carry a moderate-impact variant (OR = 0.67; *P* = .043).

**Table 2.** Frequencies of predicted deleterious *MC3R* variants in women with delayed or early menarche timing

Variant class	Menarche timing (age)	No. noncarriers	No. carriers	Carrier frequency, %	OR vs normal timing	95% CI	*P*
High	Delayed (≥16 y)	23 216	13	0.0560	2.3466	1.17-4.38	.0098
High	Early (<10 y)	9321	2	0.0215	0.8992	0.11-3.42	≥.999
High	Normal	213 725	51	0.0239	—	—	—
Moderate excl. benign	Delayed (≥16 y)	23 088	141	0.6070	1.5155	1.26-1.81	1.45E-05
Moderate excl. benign	Early (<10 y)	9298	25	0.2682	0.6672	0.43-0.99	.0430
Moderate excl. benign	Normal	212 918	858	0.4014	—		—
Moderate/high excl. benign	Delayed (≥16 y)	23 076	153	0.6587	1.5527	1.30-1.85	1.77E-06
Moderate/high excl. benign	Early (<10 y)	9296	27	0.2896	0.6802	0.45-1.00	.0489
Moderate/high excl. benign	Normal	212 867	909	0.4252	—	—	*—*
CADD ≥25	Delayed (≥16 y)	23 097	132	0.5683	1.6633	1.37-2.01	3.90E-07
CADD ≥25	Early (<10 y)	9300	23	0.2467	0.7198	0.45-1.09	.1437
CADD ≥25	Normal	213 044	732	0.3424	—	—	*—*

Data are from the UK Biobank 450k exome cohort. ORs were calculated for frequency of carriers for *MC3R* coding variants in clinically defined categories of self-reported menarche timing. Variant classes were defined by variant effect predictor: high = protein-truncating variants; moderate = missense variants/in-frame insertion/deletions. Benign variants were excluded as classified by SIFT/Polyphen. CADD score cutoff set to 25 or greater.

Abbreviations: CADD, Combined Annotation-Dependent Depletion; *MC3R*, melanocortin 3 receptor; OR, odds ratio.

While providing a precise estimate for pubertal onset is more challenging in males, the 190 547 men in UK Biobank who reported an “older than average” age at voice-breaking were 1.6-fold more likely to carry a moderate-impact *MC3R* variant compared to men with an average age at voice-breaking (*P* = 7.46E-04) (Supplementary Table S4 (26)).

## Discussion

These data provide evidence suggesting that deleterious mutations in *MC3R* are more frequently found in patients presenting with CDGP but not in those with nIHH. Although the overall number of *MC3R* variants identified was small, previous studies investigating the presence of LoF variants in hypothalamic-pituitary-gonadal genes in patients with self-limited DP report only small numbers of patients who carry a variant in any individual gene (8-10, 12, 35).

In the population-based UK Biobank cohort, we observed an increased frequency of predicted damaging *MC3R* variants in women with delayed compared to normal age at menarche. This result is concordant with a previous association of rare, predicted deleterious *MC3R* variants with later age at menarche when assessed as a continuous variable in the smaller 200k UK Biobank exome data release (15). The majority of carriers of high-impact *MC3R* variants still report “normal” timing of menarche, evidence that *MC3R* haploinsufficiency has a low level of penetrance and likely influences pubertal timing within a wider polygenic and environmental context.

We also observed a lower frequency of predicted damaging *MC3R* variants in those individuals with early menarche. These findings suggest that MC3R tone may be relevant to both extremes of the population’s distribution of pubertal timing. It is still unknown whether MC3R activation alone can promote early pubertal timing. Future discovery and characterization of the effect of *MC3R* gain-of-function variants on pubertal timing and reproductive function would be informative.

We found that deleterious *MC3R* variants are infrequent and not overrepresented among patients with nIHH. This finding is consistent with the evidence that MC3R is a permissive rather than an essential factor for pubertal timing; *Mc3r* knockout mice still progress through puberty, albeit with significant delay, and the reported male homozygote carrier of *MC3R* LoF variant also eventually progressed through puberty to achieve reproductive capacity (15).

We acknowledge limitations of this study, including the lack of matched control groups to account for the ancestry of patients and sequencing coverage. As most patients are of European ancestry, we used ALSPAC as a control group with an approximately equivalent genetic background. Exclusion of patients with non-European ancestry within the DPGen cohort did not change the overall frequency of LoF *MC3R* carriers, indicating this overrepresentation is not confounded by ancestry differences. The growing body of genetic and phenotypic information on cohorts of non-European ancestry will, in time, allow more robust testing of the role of rare variants in *MC3R* in CDGP across different populations. The 171 *MC3R* variants found in the UK Biobank were classified based on their predicted deleteriousness rather than in vitro functional assays because of the large number of variants found; a more accurate estimate of LoF carriage will require a high-throughput approach for functionally characterizing all *MC3R* protein-altering variants within this cohort.

The reproductive role of MC3R is tightly linked to whole-body nutritional status as MC3R activation is regulated by the leptin-proopiomelanocortin pathway, and *MC3R* variants are also associated with shorter stature, lower circulating insulin-like growth factor-1, and reduced indices of lean mass (15, 36). While the focus of this study was DP diagnoses, future prospective studies that include a systematic measure of these phenotypes in patients with DP would be valuable in ascertaining if patients carrying *MC3R* variants also exhibit nutritionally related phenotypes.

In conclusion, *MC3R* LoF variants are more frequently found in patients presenting with CDGP, and within the general population predicted damaging variants are more common in those with self-reported DP compared to normally timed puberty.

## Data Availability

All data used in the UKB association analysis are available from the UKB on application (https://www.ukbiobank.ac.uk/). This study has been conducted under UK Biobank application number 9905. The ALSPAC study website contains details of all the data that are available through a fully searchable data dictionary and variable search tool (https://www.bristol.ac.uk/alspac/researchers/our-data/). This study used data under project numbers B2891 and B4033. Some data sets analyzed during the present study are not publicly available but are available from the corresponding authors on reasonable request.

## Abbreviations

ALSPAC: Avon Longitudinal Study of Parents and Children
CADD: Combined Annotation-Dependent Depletion
cAMP: cyclic adenosine monophosphate
CDGP: constitutional delay of growth and puberty
CLoF: complete loss-of-function
DP: delayed puberty
DPGen: Delayed Puberty Genetic Consortium
HH: hypogonadotropic hypogonadism
HRC: Haplotype Reference Consortium
LoF: loss-of-function
*MC3R*: melanocortin 3 receptor
nIHH: normosmic idiopathic hypogonadotropic hypogonadism
OR: odds ratio
PLoF: partial loss-of-function
VEP: variant effect predictor
WES: whole-exome sequence
WT: wild-type
